# Empyema in Childhood

**Published:** 1902-10-04

**Authors:** 


### EMPYEMA IN CHILDHOOD.

Dr. Henry Koplik,[1] of New York, writing on the subject of empyema, says that it is not possible in infants and children to sharply differentiate the symptoms of pleurisy with effusion from those of empyema. They merge into one another, but fully 40 per cent. of the pleurisies occurring in infancy and childhood are purulent, while in adults only 5 per cent. of pleurisies are of this nature. Etiologically, empyema follows some acute affection of the lung in fully 95 per cent. of the cases in infants and children, and in a majority of such cases this precedent condition was probably a pneumonia pure and simple, the pneumococcus being found in the exudate either in pure culture or combined with the streptococcus pyogenes. On the other hand, the tuberculous forms of empyema are the most infrequent variety in children, although in adults fully

45 per cent. of the pleurisies, including empyema, are tuberculous.

In infants and children empyema is an acute affection following all forms of pneumonia, the infectious diseases, such as measles, scarlet fever, etc., certain forms of tonsillitis, retropharyngeal abscess, chronic intestinal catarrh, sepsis of the newborn, osteomyelitis and appendicitis. In the majority of cases the purulent effusion into the pleura has the gross characteristics of ordinary pus. In other cases the effusion is first a clear serous effusion, and subsequently is seen to change into a purulent one without any extraneous infection. In very rare cases the exudation into the chest is hæmorrhagic; but hæmorrhagic empyema does not have the serious significance in children that it has in the adult—it is not necessarily tuberculous or due to a new growth of the pleura.

As for symptoms the disease is apt to be masked by those of the main causal affection; but when this has run its course and the temperature has dropped to normal and has perhaps continued so for a few days, a slight rise of temperature may point to the presence of fluid in the chest, and puncture at this period may indicate a serous effusion. In such cases, however, the temperature may show a sudden rise of two or more degrees at the end of the second week, and puncture then will disclose that the effusion has become purulent.

In other cases the temperature, pulse, and respiration, after the first week or ten days of the pneumonia, do not recede to the normal. There is a persistent temperature with increased pulse and rapid respiration, while the signs in the chest, instead of clearing up, only become more and more marked, resembling very closely an extension of the pneumonia, when in reality there is, in addition to the process in the lung, fluid in the pleura. Puncture after twelve to fourteen days reveals an empyema.

In other cases, again, the empyema seems to run side by side with the pneumonia, so that at the eighth day signs of effusion may already be found.

In still another series of cases the progress of the disease may be more insidious. After an attack of fever there remains a slight hacking cough, emaciation, pallor, and loss of appetite, and to these may be added night sweats. On examining the chest in such a case the pleura may be found full of fluid.

No diagnosis of this disease is complete without exploratory puncture of the chest. This is a comparatively simple procedure, devoid of any danger. It may be said that, rather than be in doubt, we should enter the exploring needle into any chest in which the signs lead us to suspect fluid. With ordinary care we do not subject the patient to any risk of further infection. We cannot readily cause a serous effusion to become purulent if ordinary cleanliness is exercised. Puncture should always be made with the patient in the upright position. One or two punctures only should be made at a sitting, but if no fluid is obtained, we should not hesitate to repeat the process, after a reasonable interval, should signs and symptoms persist. After operation, whether by incision or resection, the complication most to be feared is bronchopneumonia, generally on the affected but sometimes on the unaffected side. In children the outlook in tuberculous empyema is, on the whole, much better than in the adult subject.

[1] New York Medical News.

